# Sexually transmitted hepatitis C virus infections: current trends, and recent advances in understanding the spread in men who have sex with men

**DOI:** 10.1002/jia2.25348

**Published:** 2019-08-30

**Authors:** Bernadien M Nijmeijer, Jelle Koopsen, Janke Schinkel, Maria Prins, Teunis BH Geijtenbeek

**Affiliations:** ^1^ Department of Experimental Immunology Amsterdam Infection and Immunity Institute Amsterdam University Medical Centers University of Amsterdam Amsterdam The Netherlands; ^2^ Department of Medical Microbiology Laboratory of Clinical Virology Amsterdam Infection and Immunity Institute Amsterdam University Medical Centers University of Amsterdam Amsterdam The Netherlands; ^3^ Department of Infectious Diseases, Research and Prevention Public Health Service of Amsterdam Amsterdam The Netherlands

**Keywords:** hepatitis C virus, sexual transmission, men who have sex with men, epidemiology, dendritic cells, prevention

## Abstract

**Introduction:**

Hepatitis C virus (HCV) is a major public health threat. Although the recent availability of highly effective directly acting antivirals created optimism towards HCV elimination, there is ongoing transmission of HCV in men who have sex with men (MSM). We here report current epidemiological trends and synthesise evidence on behavioural, network, cellular and molecular host factors associated with sexual transmission of HCV, in particular the role of HIV‐1 co‐infection. We discuss prevention opportunities focusing on the potential of HCV treatment.

**Methods:**

We searched MEDLINE, fact sheets from health professional bodies and conference abstracts using appropriate keywords to identify and select relevant reports.

**Results and discussion:**

Recent studies strongly suggest that HCV is transmitted via sexual contact in HIV‐positive MSM and more recently in HIV‐negative MSM eligible for or on pre‐exposure prophylaxis. The reinfection risk following clearance is about 10 times the risk of primary infection. International connectedness of MSM transmission networks might contribute to ongoing reinfection. Some of these networks might overlap with networks of people who inject drugs. Although, the precise mechanisms facilitating sexual transmission remain unclear, damage to the mucosal barrier in the rectum could increase susceptibility. Mucosal dendritic cell subsets could increase HCV susceptibility by retaining HCV and transmitting the virus to other cells, allowing egress into blood and liver. Early identification of new HCV infections is important to prevent onward transmission, but early diagnosis of acute HCV infection and prompt treatment is hampered by the slow rate of HCV antibody seroconversion, which in rare cases may take more than a year. Novel tests such as testing for HCV core antigen might facilitate early diagnosis.

**Conclusions:**

High‐risk sexual behaviour, network characteristics, co‐infection with sexually transmitted infections like HIV‐1 and other concomitant bacterial and viral sexually transmitted infections are important factors that lead to HCV spread. Targeted and combined prevention efforts including effective behavioural interventions and scale‐up of HCV testing and treatment are required to halt HCV transmission in MSM.

## 1 Introduction

In 2015, viral hepatitis was responsible for an estimated 1.3 million deaths from acute infection and hepatitis‐related liver cancer and cirrhosis – a toll comparable to that of HIV and tuberculosis 1. Hepatitis C virus (HCV) infections account for almost 30% of these deaths. Worldwide most HCV infections have been acquired by exposure to infected blood or blood products. After the first commercial test became available in 1991 and HCV transmission through blood product was effectively halted, sharing of injecting equipment among people who inject drugs (PWID) became the major route of transmission in high‐income countries 2. In contrast to hepatitis B, the risk of sexual transmission of HCV has always been considered low 3, 4. This low risk was confirmed by a recent study among 500 anti‐HCV‐positive, HIV‐negative persons and their long‐term HCV‐negative heterosexual partners, reporting a maximum incidence rate of HCV transmission by sex of 0.07% per year or one infection per 190,000 sexual contact, and a lack of association with specific sexual practices 5. However, in the mid‐2000s, HCV infection emerged in men who have sex with men (MSM) 6, likely due to sexual contact 7. Although there was skepticism among some investigators, who assumed the cause was underreporting of injecting drugs, further evidence from Europe, the United States and Australia that MSM who denied injecting drug acquired HCV 8, 9, reopened the discussion on the importance of sexual transmission of HCV 7. The high reinfection rates among MSM who cleared HCV spontaneously or who were successfully treated 10, 11, 12, further underscored the importance of sexual behaviour in HCV transmission. As new HCV infections were typically found in HIV‐positive MSM, it was initially suggested that HIV‐1 status could be an important factor for sexually acquired HCV 10, 13, 14, 15. However, recent studies suggest that sexual transmission of HCV also occurs in HIV‐1‐negative MSM eligible for or using pre‐exposure prophylaxis (PrEP), indicating that HIV‐1 infection status is not the only factor affecting susceptibility 16, 17, 18. The frequency of exposure to HCV within specific sexual networks is also important as recent studies show that HIV‐negative MSM are infected with HCV‐strains already circulating among HIV‐positive MSM 19, 20, 21. Although directly acting antiviral (DAA) treatment is very effective in clearing HCV 22, and its availability created optimism towards HCV elimination, the high HCV (re)infection rates, likely via sexual contact, highlight the need for a better understanding of the mechanisms involved in sexual transmission of HCV.

We reviewed the current knowledge regarding HCV infection in MSM to summarize epidemiological trends and synthesise evidence on behavioural, network and host factors associated with sexual transmission of HCV. We also discuss prevention opportunities focusing on the potential of HCV infection treatment programmes on the spread of sexually acquired HCV.

## 2 Methods

We have systemically searched MEDLINE, fact sheets from health professional bodies including the World Health Organization, Center for disease Control and Prevention, the American Association for the Study of Liver Diseases and recent conference abstracts, published in English before January 2019. We have searched these databases using the following keywords: HCV, acute HCV, sexual transmission, MSM, HIV‐1 coinfection, DAA, PrEP, reinfection, molecular epidemiology, HCV diagnosis, HCV treatment guidelines, phylogenetics and phylogeography to identify and select relevant reports.

### 2.1 Epidemiology of sexually transmitted HCV

#### 2.1.1 Trends in HCV infections in HIV‐positive and ‐negative MSM

Outbreaks of sexually transmitted HCV have been reported globally among HIV‐positive MSM since 2000 7, 23. Using data from the international CASCADE collaboration, it was found that HCV incidence among HIV‐positive MSM significantly increased from 0.07/100 person‐years in 1990 to 1.8 per 100 person years in 2014 24. These findings are in line with the incidence rates and the time trend observed in a meta‐analysis pooling incidence data from 17 individual studies 10. Trends differed per European region: while HCV incidence has stabilized in western Europe, likely due to increased awareness, testing and uptake of therapy, it continues to increase in northern Europe 24. Furthermore, time from HIV to HCV infection has shortened in recent years 24. The risk of reinfection is more than 10 times higher than primary infections, which is of great concern 10. The European NEAT study, including data from eight centres in Austria, France, Germany and the UK, reported an overall reinfection incidence of 7.3/100 person‐years in HIV‐positive MSM who spontaneously cleared their HCV infection, which occurs in approximately 15% of acute HCV infections in HIV‐positive MSM 25, or responded to treatment 12. These findings are in line with studies from Australia and elsewhere in Europe, showing that up to one‐third acquired a reinfection within two years 11, 26, 27, 28. Temporal trends in the incidence of HCV reinfection have not been investigated, with exception of one recent study from Canada showing that reinfection rates did not diminish over time 29. Reinfection rates in this study were about half the rates observed in studies from Europe and Australia, indicating that infection rates might be regional specific 29.

In contrast to HIV‐positive MSM, HIV‐negative MSM are generally not in routine clinical care. Hence, data on HCV incidence are more difficult to obtain. Meta‐analyses estimated a 4‐to‐19‐fold times lower HCV incidence in HIV‐negative MSM compared to their HIV‐positive counterparts and a pooled incidence rate of 0.04‐0.15/100 person‐years in HIV‐negative MSM 30, 31, 32. This is comparable to the incidence observed among HIV‐positive MSM in the early 1990s 10, 24. The HCV prevalence among HIV‐negative MSM ranged between 0.3% and 1.5% in studies published from 2012 to 2018 33, 34, 35, 36, 37, 38, 39, 40. These data suggest that HV‐negative men remain largely unaffected by the outbreak of HCV among HIV‐positive MSM. A higher prevalence (3‐4%) was found in studies from Canada and the U.S., but HCV infections were strongly associated with lifetime injecting drug use 41, 42. Data on a rise in HCV incidence among HIV‐negative MSM are limited and inconsistent 7. A serial cross‐sectional study among HIV‐negative MSM attending a large clinic treating sexually transmitted infections (STI) in the Netherlands showed a stable HCV prevalence (about 1% each year) over the period 2007‐2017 39, suggesting HCV incidence is not increasing in this group. Recently, an unexpectedly relatively high anti‐HCV prevalence (4.8%) was found at PrEP initiation among MSM enrolled in a PrEP demonstration project in the Netherlands 19. An additional concern is that during follow‐up in PrEP studies in France and the Netherlands, HCV incidence rates of about 1/100 person‐years for primary HCV infection [20,43] and 25/100 person‐years for reinfection were found 43, comparable to incidence rates for HIV‐positive MSM. Acute HCV infections in MSM using PrEP have also been reported in the United States and United Kingdom 17, 18.

#### 2.1.2 Molecular epidemiology

Molecular epidemiology is increasingly used to identify clusters and transmission pathways in rapidly evolving pathogens such as HIV and HCV. The main aim of these molecular approaches was to aid the public health response by identifying factors of the epidemic, such as hotspots or emerging clusters, otherwise missed.

Molecular epidemiology has revealed several important aspects of the complexity of HCV transmission networks since the first reports on sexually transmitted HCV infections were published in the mid‐2000s. Phylogenetic analyses of HCV sequences derived from HIV‐positive MSM in England, the Netherlands, Germany, France 23, 44, Australia 45 and the USA 46 between 2002 and 2009 revealed the international connectedness of transmission networks. Molecular approaches also demonstrate the overlap of MSM and PWID clusters in Australia, suggesting the existence of social networks in which both injection drug use and sexual risk behaviours are present 47. The opposite has also been observed: no overlap of MSM and PWID was observed in the Netherlands when comparing genotype 4 infections 48. Hence, geographically distinct clustering patterns exist. Transmission clusters of genotypes 1a, 1b, 3a and 4d in MSM have been described globally and represent the major circulating variants, although regional differences exist. In Australia, genotype 1 and 3 are overrepresented among MSM, whereas in the United States subtypes 1a and 1b are more prevalent 40. Subtypes 1a and 4d cause the majority of infections among MSM in western Europe 12, 23, whereas in Asia, subtype 1b and 3a are more prevalent 23, 49, 50. Moreover, subtype distribution may even vary by country.

Molecular sequence analyses have demonstrated that HIV‐negative MSM on PrEP or eligible for PrEP in the Netherlands and France are infected with HCV strains circulating among HIV‐positive MSM 19, 43. Transmission from HIV‐positive to HIV‐negative MSM seems to occur 19, 21. It is difficult to determine precisely to what extent this transmission occurs via injecting drug use, sexual transmission, or other risk factors, but it seems unlikely that injecting drug use is responsible for a majority of the transmission events in HIV‐negative MSM; of the HCV‐positive MSM using PrEP in the Amsterdam PrEP cohort, only 23.5% (4/18) reported injecting drug use 19, but in France this was 83% (5/6) 21. However, numbers in both studies were small. Furthermore, declaring injecting drug use does not equate to sharing injection equipment. Viral sequences collected in Australia and New Zealand suggest that HCV transmission occurs through discrete networks, particularly among HIV and HCV co‐infected individuals 51. In this study, three distinct risk profiles based on the molecular analysis were described: PWID, HIV‐positive MSM with low probability of injecting drug use, and MSM with both injecting drug use and sexual risk behaviour. Some clusters with low‐probability of injecting drug use contained both HIV‐positive and HIV‐negative MSM.

These findings suggest that sexual networks of HIV‐positive and HIV‐negative overlap and that HCV transmission occurs between the two groups. Molecular analyses of already collected HCV strains provide insight in the network complexities of sexual HCV transmission. However, they do not easily translate into actionable public health interventions. Real‐time molecular surveillance of these networks may be necessary to eliminate HCV from local MSM communities, especially since high HCV treatment uptake may not be sufficient to lower the HCV incidence in this population, as shown in France 52. Monitoring of cluster emergence, cluster growth, and cluster characteristics provides a way to identify an outbreak early and the drivers thereof. For HIV, efforts to develop such a system led to HIV‐TRACE, a real‐time molecular surveillance tool that produces data that can be translated into action 53, 54. Real‐time molecular surveillance could aid public health professionals in focusing prevention efforts; an epidemic with new infections that primarily cluster with other locally circulating variants requires a different prevention approach than an epidemic with mostly externally introduced variants. In order to facilitate characterization of external introductions, good regional or global reference sequences are necessary, and testing in combination with active data sharing of HCV sequences is needed. Lastly, network variables that may correlate with cluster emergence/growth (e.g. venue of meeting sexual partners, belonging to specific subcultures) 55, 56 should be collected prospectively to target specific prevention measures.

#### 2.1.3 Risk factors for acquiring sexually transmitted HCV

Evidence on risk factors for acute HCV infection is largely based on studies among HIV‐positive MSM evaluating determinants of primary HCV infection. Although study design, statistical approach and data collection on potential risk factors differ across studies, these studies have consistently shown that in multivariable analyses incident or acute HCV infection is associated with high risk sexual behaviour, including receptive condomless anal intercourse, unprotected fisting, sharing of toys, chemsex and group sex 10, 31, 57, 58, 59, 60. Also, the association with recent STIs supports a sexual route of HCV transmission 13, 58, 60, 61, 62, 63, 64. In addition, a recent study from Canada concluded that all but one HCV reinfection in MSM appeared to have been sexually transmitted 29 and the few studies that restricted behavioural risk factor analysis to MSM who denied injecting drug use, demonstrated risks of sexual transmission of HCV 8, 65. However, there is also evidence for blood‐to‐blood routes of HCV transmission: injecting drug use, which is reported by a minority of HCV‐positive MSM in several studies, sharing snorting drug equipment (straws) and rectal bleeding are associated with an increased risk of incident HCV infection 57, 58, 60, 66, 67, 68. Furthermore, younger MSM, peaking at around age 35, are at increased risk of incident HCV infection 24, 62.

Finally, studies consistently show that biological factors might play a role: confection with STI, HIV‐1 infection in itself, a lower CD4 cell count and higher HIV RNA levels are associated with an increased risk of incident HCV infection 24, 58, 66, 68. These factors might affect the mucosal microenvironment and activate specific immune cells within mucosal tissues, which would allow HCV entry and retention.

### 2.2 Dendritic cells in sexual transmission of HCV

**Figure 1:**
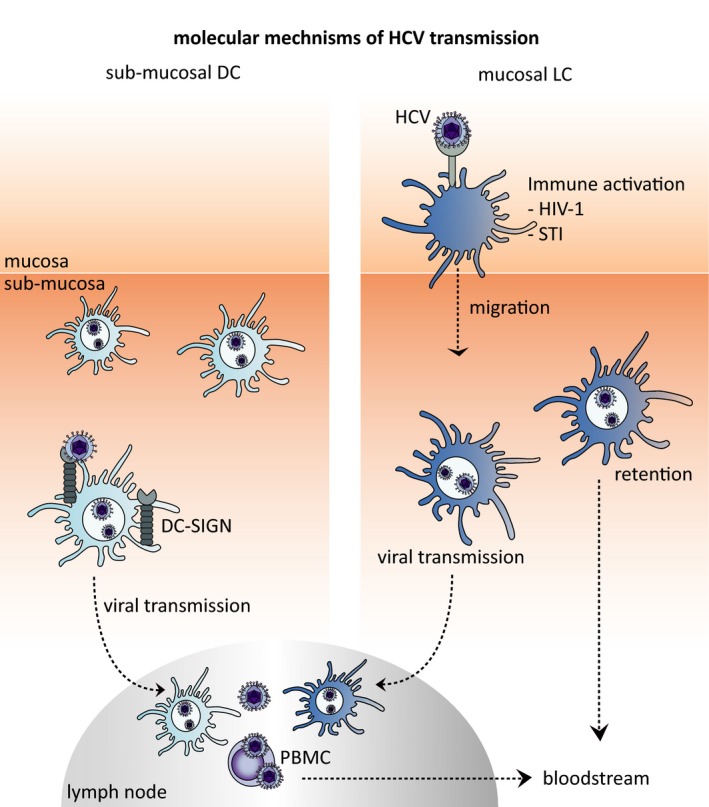
Molecular mechanisms of HCV transmission (**A**) Sub‐mucosal DCs capture HCV and migrate into the lymphoid tissues to transmit HCV to PBMCs which might lead to further dissemination HCV to the liver. (**B**) Mucosal LCs capture HCV after immune activation by STIs and either retain HCV in the tissue which could increase the chance of virus to egress into the bloodstream and disseminate to the liver or migrate into the lymphoid tissues thereby allowing HCV dissemination to the liver. DC‐SIGN, dendritic cell‐specific ICAM‐grabbing non‐integrin; HCV, Hepatitis C virus; HIV‐1, Human immunodeficiency virus type 1; PBMC, peripheral blood mononuclear cells; STI, Sexual transmitted infections.

HCV coinfections with other STIs such as HIV‐1, Herpes Simplex Virus type 2 (HSV‐2), Chlamydia, Human Papillomavirus (HPV), gonorrhoea and syphilis are common 69, 70, 71, suggesting that STIs might directly affect the increased susceptibility to HCV upon sexual contact. Dendritic cell (DC) subsets play an important role in sexual transmission of viruses such as HIV‐1 and HCV across mucosal tissues 72, 73. DCs patrol the mucosal tissues to capture invading pathogens for antigen presentation to T cells in the lymph nodes 74. Anal intercourse is the primary route for HIV‐1 infection among MSM individuals 75, underscoring the importance of the anal mucosa as entry site for sexually transmitted viruses. Langerhans cells (LCs), a mucosal DC subset, have been identified in human sigmoid colon, rectal mucosal tissues 76 and anal tissue of MSM 73, 77, 78, 79. Also, HCV is shed into the rectum of MSM with HCV infection 80. Therefore, LCs could be among the first cells that encounter HCV upon sexual contact. Recently, it has been shown that immature LCs do not transmit HCV but activation of LCs changes this protective behaviour and allows for HCV dissemination to hepatocytes (Figure 1) 73. HIV‐1 infection or activation alters the ability of LCs to efficiently capture and retain infectious HCV either for transmission or to receptive cells for HCV viral egress into the bloodstream (Figure 1) 73. Also, plasmacytoid DCs (pDCs) are able to sense HCV to receptive cells resulting in antiviral type I interferon (IFN) production by pDCs 81, therefore inhibiting viral spread without becoming infected themselves 82. Both LCs and submucosal DCs migrate to lymph nodes. The migration of DCs to the lymph nodes might allow transmission of HCV to T cells, as HCV RNA has been detected in peripheral blood mononuclear cells 83, 84, 85, 86.

Various receptors have been identified on different DC subsets that are efficient in virus capture, infection and transmission 87, 88. The C‐type lectin receptors (CLRs) DC‐SIGN and L‐SIGN recognize high‐mannose N‐glycans expressed by different viruses and viral glycoproteins to promote capture of the virus through their carbohydrate recognition domain 89, 90. Both DC‐SIGN and L‐SIGN interact with HCV glycoproteins expressed by pseudotyped HCV particles or HCV present in sera of infected individuals 88, 91. Co‐culture of HCV‐treated cells with human liver cells leads to virus transmission to the susceptible liver cells *in vitro* 92, 93. Thus, DC‐SIGN and L‐SIGN mediate HCV transmission and moreover, capture by these CLRs protects the virus from degradation 94, which could further enhance HCV dissemination. L‐SIGN is expressed by liver sinusoidal endothelial cells and could therefore facilitate egress from blood into the liver 88. DC‐SIGN is expressed by sub‐mucosal DCs and could be involved in sexual transmission of HCV. Notably, single nucleotide polymorphisms in DC‐SIGN that reduce DC‐SIGN expression were shown to be associated with a reduced risk of acquiring HCV sexually within a MSM cohort 95. Upon activation, LCs might upregulate other attachment receptors that facilitate capture and transmission. Cell membrane HSPG, called Syndecans have shown to be important in HCV infection of hepatocytes 96. The interplay of attachment receptors might be important in allowing HCV entry into mucosal tissues and further dissemination of HCV to the liver. Thus, HCV might hijack DC subsets for transmission and important determinants are HIV‐1 exposure and/or immune activation by other STIs. Novel therapies targeting HCV interaction with DC subsets and abrogation of DC activation by HIV‐1 or other STIs might prevent HCV transmission.

### 2.3 Prevention and the treatment potential

Currently, there is no vaccine to prevent HCV infection. However, the recent availability of DAA for the treatment of chronic HCV with cures rates over 95% 97 has created optimism towards HCV elimination. In many countries treatment is now available for all individuals with a chronic HCV infection, irrespective of fibrosis stage 98. Modelling studies were the first to demonstrate that rapid scale‐up of DAA might limit onward transmission and chronic HCV prevalence and incidence among MSM could decline 99, 100, 101. However, for substantial reductions a decline in risk behaviour is needed as the scale‐up of DAA is counterbalanced by ongoing risk behaviour, resulting in initial and reinfections 99, 100, 101. In addition, early treatment, including treatment of acute infection, might further reduce HCV incidence 101, 102. As treatment is costly and treatment uptake varies considerably across countries 103, effective behavioural interventions for MSM at risk of (re‐)infection are urgently needed. Qualitative research among HIV‐positive MSM with a cured HCV infection in the pre‐DAA era showed that the strongest motive to implement risk reduction strategies was the reward of avoiding HCV retreatment and its side effects 104, but this may have changed with the less burdensome DAA treatment. Also sexual risk norms within the MSM population, HCV stigma and non‐disclosure of HCV status forms barriers to safer sex, and drug use directly impedes the self‐efficacy of MSM to take risk reduction measures 104.

Recently, several studies evaluating the effect of behavioural and/or testing interventions with prompt treatment, on HCV incidence among HIV‐positive MSM have been initiated 104. “Real‐life” settings in the Netherlands and Switzerland showed that high uptake of DAA among HIV‐HCV co‐infected MSM in clinical care, in Switzerland combined with intensive HCV‐RNA screening and behavioural intervention, was followed by a reduction in HCV incidence 64, 105. In Switzerland, intensive HCV‐RNA screening combined with behavioural intervention was followed by a reduction in HCV incidence 64, 105. However, in France, despite a comparable DAA uptake and cure rate, incidence of primary HCV infection continued to increase and reinfection incidence did not significantly change 52. More data from “real‐life” settings are needed to clarify the impact of DAA uptake on the epidemic. As HCV is also circulating among HIV‐negative MSM with high risk behaviour 19, 20, 52, 106 effective interventions, behavioural counselling and routine HCV testing as part of comprehensive sexual health care are needed, to curb the HCV epidemic, in particular for MSM eligible for or using PrEP. For the larger population of HIV‐negative MSM routine screening is not recommended but periodic monitoring of HCV prevalence remains important 107. Finally, efforts to identify and motivate the relatively small proportion of MSM unaware of their positive HIV‐1 status to test should be continued as this group might harbor undiagnosed HCV infections.

### 2.4 Diagnosis and testing

A large proportion of acute new HIV infections among MSM is caused by MSM who were themselves recently infected by HIV 108. However, for sexually transmitted HCV there are no studies yet formally quantifying sources of recent infections. The continuing transmission of HCV among MSM in areas with high treatment uptake 52, 64, 105 suggests that apart from undiagnosed HCV infections in MSM, recently HCV‐infected MSM might disproportionally contribute to onward transmission. For treatment as prevention to succeed, early diagnosis and prompt treatment of any new infection is paramount and testing frequency is an important factor in determining success of treatment as prevention 109, 110. Diagnosis of chronic HCV infection includes detection of anti‐HCV antibodies, followed by an HCV‐RNA test, to distinguish between past and ongoing infection. Diagnosis of acute HCV infection is more challenging as clinical signs and symptoms pointing to acute hepatitis are often absent or aspecific 111. In addition, HCV‐specific antibodies may take a long time to appear: the median time from infection to seroconversion for HCV antibodies is 74 to 91 days in HIV‐positive MSM 112, 113. In addition, a minority of patients (less than 5%) remain anti‐HCV negative for more than a year 113, 114. Delayed or even absence of seroconversion appears to be caused by HIV‐related immunosuppression, as a CD4 + count below 200 cells\μL was associated with seronegative HCV infection 115. Finally, for diagnosis of acute HCV reinfection, antibody tests cannot be used as after clearance of a primary infection, antibodies may remain present for a long time 112. Clearly, for diagnosing acute infection early, regular screening, also in asymptomatic patients with a test that directly detects viral RNA or antigen rather than antibodies would be the optimal testing strategy for identifying new cases.

As this comes with a considerable cost, measuring liver enzymes as Alanine Aminotransferase (ALT) level is frequently used as a first step in a diagnostic testing algorithm and has been shown to be more sensitive than testing for anti‐HCV antibodies for diagnosing acute HCV infection 113, 116. Although using ALT levels as a first screening step greatly reduces cost as compared to directly detecting HCV RNA, this may result in early acute cases remaining undiagnosed 112, 116.

Recently, HCV core antigen has been shown to be a reliable marker for diagnosing HCV infection in chronically infected patients 117. Regular screening for HCV core antigen may therefore present an attractive strategy for frequent screening of MSM at risk for sexually transmitted HCV. However, reported sensitivity of the core antigen test in a large study with chronically infected patients was 94% when compared with HCV RNA as a gold standard 117. The reduced sensitivity compared to HCV RNA testing, could result in acute cases remaining undiagnosed, as these sometimes present with low HCV RNA levels. The few small studies validating the core antigen test for the detection of acute HCV report a sensitivity of 89% to 100% 68, 118, 119. Larger studies which include acute HCV cases with a well‐documented narrow window of infection are needed before antigen testing can be recommended as a reliable screening strategy for acute HCV infection in routine care.

The cost‐effectiveness of HCV screening in MSM could also be increased by focussing on MSM with behaviour facilitating HCV acquisition. Indeed, according to guidelines of the American Association for the Study of Liver Diseases, men with reported high risk behaviour should be offered more frequent HCV testing than the minimal recommended annual testing frequency 109, 110. Risk behaviour can be quantified by using a risk score that is based on risk factors associated with HCV infection. A risk score for identifying acute HCV cases based on six self‐reported behavioural risk factors has been developed using data from the MOSAIC study in the Netherlands and appeared to be useful in identifying MSM at high‐risk for acute HCV‐infection 39. This risk score was validated using data from three different sources and in these validation studies from Belgium, the UK and the Netherlands, sensitivity ranged from 73% to 100% 39, 107. A risk score could therefore be used as a tool to direct testing resources.

Finally, home‐based testing represents an interesting strategy to increase test uptake among high‐risk MSM, for example, MSM with a cleared HCV infection, who are at high risk for reinfection. However, currently, only anti‐HCV antibody self‐tests are available for home‐testing, which – as explained above – are not suitable for detecting early acute primary infections or reinfections 120. Dried blood spots (DBS) collected at home which are sent to a laboratory for HCV RNA testing could be an alternative strategy to facilitate HCV RNA testing. Technically, HCV RNA can be detected on DBS with sufficient sensitivity 121. The use of home‐collected DBS for this purpose remains to be formally validated in terms of technical performance and acceptance by key‐populations including key‐populations including MSM and PWID. Core‐antigen testing on DBS has lower sensitivity and is therefore less suitable for diagnosing acute HCV infection 122.

## 3 Discussion

There is growing evidence that HCV is transmitted sexually. In the past decades this epidemic was mostly confined to HIV‐positive MSM. However, recent data show that PrEP‐using MSM are also at risk for HCV infection, presumably because there is a shared HCV transmission network of HIV‐negative and HIV‐positive MSM. The association with specific sexual practices strongly suggests that behaviour plays an important role in the ongoing epidemic among MSM. The use of drugs in a sexual context, especially injecting drugs and snorting drugs, is also a major risk factor. The implementation of biomedical HIV‐1 prevention strategies, i.e. PrEP and “U=U” (undetectable is untransmittable), might have reduced condom use, and changed sexual networks. This might result in an expanding HCV epidemic in HIV‐negative MSM as HCV is more common in HIV‐positive MSM. Hence, routine HCV testing and behavioural counselling should be part of PrEP programmes and the epidemic in the larger population of HIV‐negative MSM should be closely monitored. And even though DAAs are very effective, the high rate of reinfections further highlights the need for frequent HCV‐RNA testing and providing HCV‐risk‐reduction counselling to MSM with a history of HCV in clinical care. In addition, research into effective interventions aimed at reducing risk behaviour and preventing reinfection should be prioritized as there is a lack of evidence‐based interventions and prevention messages might not be sufficient to reduce risk behaviour. Finally, prompt HCV treatment might also contribute to a decrease in HCV prevalence and incidence, especially when combined with additional interventions as part of comprehensive sexual health services.

Factors such as receptive condomless anal intercourse, immune activation by STIs and high‐risk sexual practices (e.g. fisting) might increase susceptibility to HCV and could potentially damage the mucosal tissue and cause rectal bleeding, which would facilitate HCV infection 57, 60, 123, 124. Besides mucosal damage, the activation of mucosal LCs might also allow HCV to enter mucosal tissues and dissemination. HIV‐1 infection is a major risk factor in HCV susceptibility, partly because lower CD4 counts but also low HIV‐1 replication and immune activation might increase susceptibility. Identification of the molecular mechanisms such as the receptors involved in virus attachment might lead to therapies that prevent sexual transmission of HCV.

Early identification of any recent HCV infections and thus frequent testing of MSM reporting risk behaviour is paramount as these might feed onward transmission. Real‐time sequence collection combined with molecular phylogenetics and data collection on network characteristics could identify transmission hotspots, characterize transmission clusters, and determine the relative roles of sustained local transmission versus external introductions, all directing public health efforts to restrain the HCV epidemic among MSM.

### 3.1 Study limitations

Studies have consistently shown that the incident of acute HCV infections are associated with high risk sexual behaviour. The role of hygienic procedures (e.g. cleaning sex toys) has not been assessed in these studies but would add to our understanding. Also, no direct comparison of testing strategies, that is, comparing ALT, anti‐HCV, HCV RNA and core‐antigen longitudinally, for diagnosing acute HCV infection in patients with documented seroconversion exists. As a result, recommendations about testing strategies tend to be somewhat imprecise. Moreover, data on HCV incidence in the wider population of HIV‐negative MSM are generally scarce as these men are not in routine clinical care in contrast to HIV‐1 infected MSM and MSM using PrEP. In addition, risk factors for incident infection in HIV‐negative MSM and for reinfection in HIV positive MSM have not been studied extensively. The lack of such data limits our knowledge on the biological factors that are involved in sexual transmission of HCV. Epidemiological studies show that biological factors also play a role in increased risk of HCV infection. Coinfection with STIs might affect the mucosal microenvironment and immune activation might change the function of mucosal DC subsets. However, in vivo studies are urgently needed to understand the relevance of the immune cells in HCV transmission and to decipher the route from mucosa to liver.

As HCV (re)infection rates might be regional‐specific, more data from other parts of the world than Western Europe, North America, and Australia are needed to obtain a more detailed view of the HCV epidemic among MSM. DAAs are highly effective in curing HCV, but more data from “real‐life” settings are needed to clarify the impact of DAA uptake on the epidemic.

## 4 Conclusions

It has been established that HCV can be transmitted via sexual contact. The spread of HCV among HIV‐positive MSM in the past two decades and the recent finding of HCV infections in HIV‐negative MSM eligible or on PrEP, as well as the association with specific sexual practices, strongly suggest that behaviour plays an important role in the ongoing epidemic among MSM.

Drug use in a sexual context and biological factors as coinfection with STI and HIV‐1 also seem to play a role in facilitating HCV spread. At mucosal sites, DC subsets might play a role in HCV dissemination. Targeted and combined prevention efforts including effective behavioural interventions and scale‐up of HCV testing and treatment are required to halt HCV transmission in MSM. In addition, real‐time molecular surveillance could guide and evaluate prevention strategies.

## Competing interests

All authors declare that they have no competing interests.

## Authors’ contributions

BMN wrote the manuscript, assembled and edited the manuscript. JK wrote the manuscript. JS wrote and edited the manuscript. MP wrote, edited and reviewed the manuscript. TBHG wrote, edited and reviewed the manuscript**.**

## References

[1] World Health Organization . WHO global Hepatitis report 2017. Licens CC BY‐NA‐SA 30IGO. 2017;67.

[2] Shepard C , Finelli L , Alter M . Global epidemiology of hepatitis C virus infection. Lancet Infect Dis. 2005;5(9):558–567. 16122679 10.1016/S1473-3099(05)70216-4

[3] Osmond DH , Charlebois E , Sheppard HW , Page K , The S , Diseases I , et al. Comparison of risk factors for hepatitis C and hepatitis B virus infection in homosexual men. J Infect Dis. 1993;167(1):66–71. 8418184 10.1093/infdis/167.1.66

[4] Feldman JG , Minkoff H , Landesman S , Dehovitz J . heterosexual transmission of Hepatitis C, Hepatitis B and HIV‐1 in a sample of Inner‐City Woman. Sex Transm Dis. 1999;27(6):338–342. 10.1097/00007435-200007000-00007 10907909

[5] Terrault NA , Dodge JL , Murphy EL , Tavis JE , Kiss A , Levin TR , et al. Sexual transmission of hepatitis C virus among monogamous heterosexual couples: the HCV partners study. Hepatology. 2013;57(3):881–889. 23175457 10.1002/hep.26164 PMC4384338

[6] Wandeler G , Dufour JF , Bruggmann P , Rauch A . Hepatitis C: a changing epidemic. Swiss Med Wkly. 2015;145:1–9. 10.4414/smw.2015.14093 25658972

[7] van de Laar TJW , Matthews GV , Prins M , Danta M . Acute hepatitis C in HIV‐infected men who have sex with men: an emerging sexually transmitted infection. AIDS. 2010;24(12):1799–1812. 20601854 10.1097/QAD.0b013e32833c11a5

[8] Centers for Disease Control and Prevention (CDC) . Sexual transmission of Hepatitis C Virus among HIV‐infected Men who have sex with Men – New York City, 2005‐2010. MMWR Morb Mortal Wkly Rep. 2011;60(28):945–950. 21775948

[9] Jordan AE , Perlman DC , Neurer J , Smith DJ , Des Jarlais DC , Hagan H . Prevalence of hepatitis C virus infection among HIV+ men who have sex with men: a systematic review and meta‐analysis. Int J STD AIDS. 2017;28(2):145–159. 26826159 10.1177/0956462416630910 PMC4965334

[10] Hagan H , Jordan AE , Neurer J , Cleland CM . Incidence of sexually transmitted hepatitis C virus infection in HIV‐positive men who have sex with men. AIDS. 2015;29(17):2335–2345. 26258525 10.1097/QAD.0000000000000834 PMC4640945

[11] Lambers FAE , Prins M , Thomas X , Molenkamp R , Kwa D , Brinkman K , et al. Alarming incidence of hepatitis C virus re‐infection after treatment of sexually acquired acute hepatitis C virus infection in HIV‐infected MSM. AIDS. 2011;25(17):F21–F27. 21857492 10.1097/QAD.0b013e32834bac44

[12] Ingiliz P , Martin TC , Rodger A , Stellbrink HJ , Mauss S , Boesecke C , et al. HCV reinfection incidence and spontaneous clearance rates in HIV‐positive men who have sex with men in Western Europe. J Hepatol. 2017;66(2):282–287. 27650285 10.1016/j.jhep.2016.09.004

[13] Wandeler G , Gsponer T , Bregenzer A , Günthard HF , Clerc O , Calmy A , et al. Hepatitis C virus infections in the swiss HIV cohort study: a rapidly evolving epidemic. Clin Infect Dis. 2012;55(10):1408–1416. 22893583 10.1093/cid/cis694

[14] van de Laar TJW , van der Bij AK , Prins M , Bruisten SM , Brinkman K , Ruys TA , et al. Increase in HCV incidence among men who have sex with men in Amsterdam most likely caused by sexual transmission. J Infect Dis. 2007;196(2):230–238. 17570110 10.1086/518796

[15] Danta M , Rodger AJ . Transmission of HCV in HIV‐positive populations. Curr Opin HIV AIDS. 2011;6(6):451–458. 22001890 10.1097/COH.0b013e32834b4974

[16] Van De Laar TJW , Paxton WA , Zorgdrager F , Cornelissen M , De Vries HJC . Sexual transmission of hepatitis C virus in human immunodeficiency virus‐negative men who have sex with men: a series of case reports. Sex Transm Dis. 2011;38(2):102–104. 20706177 10.1097/OLQ.0b013e3181ec9de5

[17] Volk JE , Marcus JL , Phengrasamy T , Bradley Hare C . Incident hepatitis C virus infections among users of HIV preexposure prophylaxis in a clinical practice setting. Clin Infect Dis. 2015;60(11):1728–1729. 25694649 10.1093/cid/civ129 PMC4850931

[18] McFaul K , Maghlaoui A , Nzuruba M , Farnworth S , Foxton M , Anderson M , et al. Acute hepatitis C infection in HIV‐negative men who have sex with men. J Viral Hepat. 2015;22(6):535–538. 25412826 10.1111/jvh.12366

[19] Hoornenborg E , Achterbergh RCA , Schim Van Der Loeff MF , Davidovich U , Hogewoning A , De Vries HJC , et al. MSM starting preexposure prophylaxis are at risk of hepatitis C virus infection. AIDS. 2017;31(11):1603–1610. 28657964 10.1097/QAD.0000000000001522

[20] Cotte L , Cua E , Reynes J , Raffi F , Rey D , Delobel P , et al. Hepatitis C virus incidence in HIV‐infected and in preexposure prophylaxis (PrEP)‐using men having sex with men. Liver Int. 2018;38(10):1736–1740. 10.1111/liv.13922 29959866

[21] Charre C , Cotte L , Kramer R , Miailhes P , Godinot M , Koffi J , et al. Hepatitis C virus spread from HIV‐positive to HIV‐negative men who have sex with men. PLoS One. 2018;13(1):1–10. 10.1371/journal.pone.0190340 PMC5749770 29293630

[22] Naggie S , Curtis C , Workowski K , Ruane P , Towner WJ , Marks K , et al. Ledipasvir and sofosbuvir for HCV in patients coinfected with HIV‐1. N Engl J Med. 2015;373:705–713 26196665 10.1056/NEJMoa1501315 PMC4892372

[23] van de Laar T , Pybus O , Bruisten S , Brown D , Nelson M , Bhagani S , et al. Evidence of a large, international network of HCV transmission in HIV‐positive men who have sex with men. Gastroenterology. 2009;136(5):1609–1617. 19422083 10.1053/j.gastro.2009.02.006 PMC4260925

[24] van Santen DK , van der Helm JJ , Del Amo J , Meyer L , D'Arminio Monforte A , Price M , et al. Lack of decline in hepatitis C virus incidence among HIV‐positive men who have sex with men during 1990–2014. J Hepatol. 2017;67(2):255–262. 28412290 10.1016/j.jhep.2017.03.038

[25] Smith DJ , Jordan AE , Frank M , Hagan H . Spontaneous viral clearance of hepatitis C virus (HCV) infection among people who inject drugs (PWID) and HIV‐positive men who have sex with men (HIV+ MSM): a systematic review and meta‐analysis. BMC Infect Dis. 2016;16:471. 27595855 10.1186/s12879-016-1807-5 PMC5011802

[26] Martinello M , Grebely J , Petoumenos K , Gane E , Shaw D , Sasadeusz J , et al. HCV reinfection incidence among individuals treated for recent infection. J Viral Hepat. 2018;24(5):359–370. 10.1111/jvh.12666 PMC5400730 28027424

[27] Martin TCS , Martin NK , Hickman M , Vickerman P , Page EE , Everett R , et al. Hepatitis C virus reinfection incidence and treatment outcome among HIV‐positive MSM. AIDS. 2013;27(16):2551–2557. 23736152 10.1097/QAD.0b013e32836381cc

[28] Thomas XV , Grady BPX , Van Der Meer JTM , Ho CK , Vanhommerig JW , Rebers SP , et al. Genetic characterization of multiple hepatitis C virus infections following acute infection in HIV‐infected men who have sex with men. AIDS. 2015;29(17):2287–2295. 26258527 10.1097/QAD.0000000000000838

[29] Young J , Rossi C , Gill J , Walmsley S , Cooper C , Cox J , et al. Risk factors for hepatitis C virus reinfection after sustained virologic response in patients coinfected with HIV. Clin Infect Dis. 2017;64(9):1154–1162. 28199495 10.1093/cid/cix126 PMC5399935

[30] Yaphe S , Bozinoff N , Kyle R , Shivkumar S , Pai NP , Klein M . Incidence of acute hepatitis C virus infection among men who have sex with men with and without HIV infection: a systematic review. Sex Transm Infect. 2012;88(7):558–564. 22859499 10.1136/sextrans-2012-050566

[31] Ghisla V , Scherrer AU , Nicca D , Braun DL , Fehr JS . Incidence of hepatitis C in HIV positive and negative men who have sex with men 2000–2016: a systematic review and meta‐analysis. Infection. 2017;45(3):309–321. 28005195 10.1007/s15010-016-0975-y

[32] Jin F , Matthews GV , Grulich AE . Sexual transmission of hepatitis C virus among gay and bisexual men: a systematic review. Sex Health. 2017;14(1):28–41. 27712618 10.1071/SH16141

[33] Tseng YT , Sun HY , Chang SY , Wu CH , Liu WC , Wu PY , et al. Seroprevalence of hepatitis virus infection in men who have sex with men aged 18‐40 years in Taiwan. J Formos Med Assoc. 2012;111(8):431–438. 22939661 10.1016/j.jfma.2011.06.022

[34] Blaxhult A , Samuelson A , Ask R , Hökeberg I . Limited spread of hepatitis C among HIV‐negative men who have sex with men in Stockholm, Sweden. Int J STD AIDS. 2014;25(7):493–495. 24352124 10.1177/0956462413515192

[35] Schmidt AJ , Falcato L , Zahno B , Burri A , Regenass S , Müllhaupt B , et al. Prevalence of hepatitis C in a Swiss sample of men who have sex with men: whom to screen for HCV infection? BMC Public Health. 2014;14(1):1–11. 24393532 10.1186/1471-2458-14-3 PMC3890510

[36] Tsai JC , Hung CC , Chang SY , Liu WC , Wu CH , Su YC , et al. Increasing incidence of recent hepatitis C virus infection among persons seeking voluntary counselling and testing for HIV and sexually transmitted infections in Taiwan. BMJ Open. 2015;5(10):9–12. 10.1136/bmjopen-2015-008406 PMC4606383 26463221

[37] Wong J , Moore D , Kanters S , Buxton J , Robert W , Gustafson R , et al. Seroprevalence of hepatitis C and correlates of seropositivity among men who have sex with men in Vancouver, Canada: a cross‐sectional survey. Sex Transm Infect. 2015;91(6):430–433. 25872512 10.1136/sextrans-2014-051928

[38] Ireland G , Higgins S , Goorney B , Ward C , Ahmad S , Stewart C , et al. Evaluation of hepatitis C testing in men who have sex with men, and associated risk behaviours, in Manchester, UK. Sex Transm Infect. 2017;93(6):404–409. 28130506 10.1136/sextrans-2016-052876

[39] Newsum AM , van Rooijen MS , Kroone M , Bruisten SM , Matser A , Hogewoning A , et al. Stable low hepatitis C virus antibody prevalence among HIV‐negative MSM attending the STI outpatient clinic in Amsterdam, 2007–2017. Sex Transm Dis. 2018;45(12):1. 10.1097/OLQ.0000000000000877 30422970

[40] Van Tieu H , Laeyendecker O , Nandi V , Rose R , Fernandez R , Lynch B , et al. Prevalence and mapping of hepatitis C infections among men who have sex with men in New York City. PLoS One. 2018;13(7):1–16. 10.1371/journal.pone.0200269 PMC6051624 30020960

[41] Seaberg EC , Witt MD , Jacobson LP , Detels R , Rinaldo CR , Young S , et al. Differences in hepatitis C virus prevalence and clearance by mode of acquisition among men who have sex with men. J Viral Hepat. 2014;21(10):696–705. 25280229 10.1111/jvh.12198 PMC4187219

[42] Remis RS , Liu J , Loutfy MR , Tharao W , Rebbapragada A , Huibner S , et al. Prevalence of sexually transmitted viral and bacterial infections in HIV‐positive and HIV‐negative men who have sex with men in Toronto. PLoS One. 2016;11(7):1–16. 10.1371/journal.pone.0158090 PMC4938580 27391265

[43] Hoornenborg E , Coyer LN , Achterbergh RCA , van der Loeff MFS , Bruisten S , de Vries HJC , et al. High incidence of hepatitis C virus (re‐)infections among PrEP users in the Netherlands: implications for prevention, monitoring and treatmen. TUPDX0104 ‐. Poster Discuss Abstr. 2018.

[44] Serpaggi J , Chaix ML , Batisse D , Dupont C , Vallet‐Pichard A , Fontaine H , et al. Sexually transmitted acute infection with a clustered genotype 4 hepatitis C virus in HIV‐1‐infected men and inefficacy of early antiviral therapy. AIDS. 2006;20(2):233–240. 16511416 10.1097/01.aids.0000200541.40633.56

[45] Matthewsa GV , Hellard M , Kaldor J , Lloyd A , Dore GJ . Further evidence of HCV sexual transmission among HIV‐positive men who have sex with men: response to Danta et al.. AIDS. 2007;21(15):2112–2113. 17885306 10.1097/QAD.0b013e3282ef3873

[46] Luetkemeyer A , Hare CB , Stansell J , Tien PC , Charlesbois E , Lum P , et al. Clinical presentation and course of acute hepatitis C infection in HIV‐infected patients. J Acquir Immune Defic Syndr. 2006;41(1):31–36. 16340470 10.1097/01.qai.0000191281.77954.27 PMC4050666

[47] Matthews GV , Pham ST , Hellard M , Grebely J , Zhang L , Oon A , et al. Patterns and characteristics of hepatitis C transmission clusters among HIV‐positive and HIV‐negative individuals in the Australian trial in acute hepatitis C. Clin Infect Dis. 2011;52(6):803–811. 21282185 10.1093/cid/ciq200 PMC3106259

[48] De Bruijne J , Schinkel J , Prins M , Koekkoek SM , Aronson SJ , Van Ballegooijen MW , et al. Emergence of hepatitis C virus genotype 4: phylogenetic analysis reveals three distinct epidemiological profiles. J Clin Microbiol. 2009;47(12):3832–3838. 19794040 10.1128/JCM.01146-09 PMC2786681

[49] Lin AWC , Sridhar S , Wong KH , Lau SKP , Woo PCY . Epidemiology of sexually transmitted viral hepatitis in human immunodeficiency virus‐positive men who have sex with men in Asia. J Formos Med Assoc. 2015;114(12):1154–1161. 26375778 10.1016/j.jfma.2015.08.008

[50] Chan DP , Lin AW , Wong KH , Wong NS , Lee SS . Diverse origins of hepatitis C virus in HIV co‐infected men who have sex with men in Hong Kong Hepatitis viruses. Virol J. 2015;12(1):1–6. 26253209 10.1186/s12985-015-0355-8 PMC4528697

[51] Bartlett SR , Applegate TL , Jacka BP , Martinello M , Lamoury FMJ , Danta M , et al. A latent class approach to identify multi‐risk profiles associated with phylogenetic clustering of recent hepatitis C virus infection in Australia and New Zealand from 2004 to 2015. J Int AIDS Soc. 2019;22(2):1–11. 10.1002/jia2.25222 PMC6371014 30746864

[52] Pradat P , Huleux T , Raffi F , Delobel P , Valantin MA , Poizot‐Martin I , et al. Incidence of new hepatitis C virus infection is still increasing in French MSM living with HIV. AIDS. 2018;32(8):1077–1082. 29438195 10.1097/QAD.0000000000001789

[53] Poon AFY , Gustafson R , Daly P , Zerr L , Demlow SE , Woods CK , et al. Near real‐time monitoring of HIV transmission hotspots from routine HIV genotyping; an implementation case study. Lancet HIV. 2016;3(5):1–15. 10.1016/S2352-3018(16)00046-1 PMC4853759 27126490

[54] Kosakovsky Pond SL , Weaver S , Leigh Brown AJ , Wertheim JO . HIV‐TRACE (TRAnsmission Cluster Engine): a tool for large scale molecular epidemiology of HIV‐1 and other rapidly evolving pathogens. Mol Biol Evol. 2018;35(7):1812–1819. 29401317 10.1093/molbev/msy016 PMC5995201

[55] Bradshaw D , Raghwani J , Jacka B , Sacks‐Davis R , Lamoury F , Down I , et al. Venue‐based networks may underpin HCV transmissions amongst HIV‐infected gay and bisexual men. PLoS One. 2016;11(9):1–16. 10.1371/journal.pone.0162002 PMC5008823 27584149

[56] Matser A , Vanhommerig J , Schim van der Loeff MF , Geskus RB , de Vries HJC , Prins JM , et al. HIV‐infected men who have sex with men who identify themselves as belonging to subcultures are at increased risk for hepatitis C infection. PLoS One. 2013;8(3):e57740. 23469226 10.1371/journal.pone.0057740 PMC3587624

[57] Schmidt AJ , Rockstroh JK , Vogel M , An der Heiden M , Baillot A , Krznaric I , et al. Trouble with bleeding: risk factors for acute hepatitis C among HIV‐positive gay men from Germany‐A case‐control study. PLoS One. 2011;6(3):28–32. 10.1371/journal.pone.0017781 PMC3050932 21408083

[58] Witt MD , Seaberg EC , Darilay A , Young S , Badri S , Rinaldo CR , et al. Incident hepatitis C virus infection in men who have sex with men: a prospective cohort analysis, 1984–2011. Clin Infect Dis. 2013;57(1):77–84. 23532480 10.1093/cid/cit197 PMC3669529

[59] Danta M , Brown D , Bhagani S , Pybus OG , Sabin CA , Nelson M , et al. Recent epidemic of acute hepatitis C virus in HIV‐positive men who have sex with men linked to high‐risk sexual behaviours. AIDS. 2007;21(8):983–991. 17457092 10.1097/QAD.0b013e3281053a0c

[60] van Hommerig JW , Lambers FAE , Schinkel J , Geskus RB , Arends JE , van der Laar TJ , et al. Risk factors for sexual transmission of Hepatitis C virus among human immunodeficiency virus‐infected men who have sex with men: a case‐control study. Open Forum Infect Dis. 2015;2:1–8. 10.1093/ofid/ofv115 PMC4665384 26634219

[61] Sun HY , Chang SY , Yang ZY , Lu CL , Wu H , Yeh CC , et al. Recent hepatitis C virus infections in HIV‐infected patients in Taiwan: incidence and risk factors. J Clin Microbiol. 2012;50(3):781–787. 22189113 10.1128/JCM.06014-11 PMC3295121

[62] Medland NA , Chow EPF , Bradshaw CS , Read THR , Sasadeusz JJ , Fairley CK . Predictors and incidence of sexually transmitted Hepatitis C virus infection in HIV positive men who have sex with men. BMC Infect Dis. 2017;17:(1):185. 28253838 10.1186/s12879-017-2288-x PMC5335771

[63] Burchell AN , Gardner SL , Mazzulli T , Manno M , Raboud J , Allen VG , et al. Hepatitis C virus seroconversion among HIV‐positive men who have sex with men with no history of injection drug use: results from a clinical HIV cohort. Can J Infect Dis Med Microbiol. 2015;26(1):17–22. 25798149 10.1155/2015/689671 PMC4353264

[64] Braun DL , Hampel B , Martin E , Kouyos R , Kusejko K , Grube C , et al. High number of potential transmitters revealed in a population‐based systematic hepatitis C virus RNA infected men who have sex with men screening among human immunodeficiency virus. Clin Infect Dis. 2018;68:561–568. 10.1093/cid/ciy545 30107494

[65] Rauch A , Martin M , Weber R , Hirschel B , Tarr PE , Bucher HC , et al. Unsafe sex and increased incidence of hepatitis c virus infection among HIV‐infected men who have sex with men: the swiss HIV cohort study. Clin Infect Dis. 2005;41(3):395–402. 16007539 10.1086/431486

[66] Taylor LE , Holubar M , Wu K , Bosch RJ , Wyles DL , Davis JA , et al. Incident hepatitis C virus infection among US HIV‐infected men enrolled in clinical trials. Clin Infect Dis. 2011;52(6):812–818. 21282184 10.1093/cid/ciq201 PMC3106260

[67] Nishijima T , Shimbo T , Komatsu H , Hamada Y , Gatanaga H , Oka S . Incidence and risk factors for incident hepatitis C infection among men who have sex with men with HIV‐1 infection in a large urban HIV clinic in Tokyo. J Acquir Immune Defic Syndr. 2014;65(2):213–217. 24185533 10.1097/QAI.0000000000000044

[68] Vanhommerig JW , Lambers FAE , Schinkel J , Geskus RB , Arends JE , van de Laar TJW , et al. Risk factors for sexual transmission of hepatitis C virus among human immunodeficiency virus‐infected men who have sex with men: a case‐control study. Open Forum Infect Dis. 2015;2(3):115. 10.1093/ofid/ofv115 PMC4665384 26634219

[69] Johnson LF , Lewis DA . The effect of genital tract infections on HIV‐1 shedding in the genital tract: a systematic review and meta‐analysis. Sex Transm Dis. 2008;35(11):946–959. 18685546 10.1097/OLQ.0b013e3181812d15

[70] Kent CK , Chaw JK , Wong W , Liska S , Gibson S , Hubbard G , et al. Prevalence of rectal, urethral, and pharyngeal chlamydia and gonorrhea detected in 2 clinical settings among men who have sex with men: San Francisco, California, 2003. Clin Infect Dis. 2005;41(1):67–74. 15937765 10.1086/430704

[71] Li X , Li M , Yang Y , Zhong X , Feng B , Xin H , et al. Anal HPV/HIV co‐infection among men who have sex with men: a cross‐sectional survey from three cities in China. Sci Rep. 2016;6:1–9. 26892938 10.1038/srep21368 PMC4759533

[72] De Jong MAWP , De Witte L , Oudhoff MJ , Gringhuis SI , Gallay P , Geijtenbeek TBH . TNF‐a and TLR agonists increase susceptibility to HIV‐1 transmission by human Langerhans cells ex vivo. J Clin Invest. 2008;118(10):3440–3452. 18776939 10.1172/JCI34721 PMC2528910

[73] Nijmeijer B , Sarrami‐Forooshani R , Steba G , Schreurs R , Koekkoek S , Molenkamp R , et al. HIV‐1 exposure and immune activation enhance sexual transmission of Hepatitis C virus by primary Langerhans cells. Int J AIDS Soc. 2019;1–9. 10.1002/jia2.25268 PMC6442005 30932366

[74] Banchereau J , Steinman RM . Dendritic cells and the control of immunity. Nature. 1998;392(6673):245–252. 9521319 10.1038/32588

[75] Baggaley RF , White RG , Boily MC . HIV transmission risk through anal intercourse: systematic review, meta‐analysis and implications for HIV prevention. Int J Epidemiol. 2010;39(4):1048–1063. 20406794 10.1093/ije/dyq057 PMC2929353

[76] Preza GC , Tanner K , Elliott J , Yang OO , Anton PA , Ochoa M‐T . Antigen‐presenting cell candidates for HIV‐1 transmission in human distal colonic mucosa defined by CD207 dendritic cells and CD209 macrophages. AIDS Res Hum Retroviruses. 2014;30(3):241–249. 24134315 10.1089/aid.2013.0145 PMC3938918

[77] Sobhani I , Walker F , Aparicio T , Abramowitz L , Henin D , Cremieux AC , et al. Effect of anal epidermoid cancer‐related viruses on the dendritic (Langerhans’) cells of the human anal mucosa. Clin Cancer Res. 2002;8(9):2862–2869. 12231528

[78] Omine Y , Hinata N , Yamamoto M , Kasahara M , Matsunaga S , Murakami G , et al. Regional differences in the density of Langerhans cells, CD8‐positive T lymphocytes and CD68‐positive macrophages: a preliminary study using elderly donated cadavers. Anat Cell Biol. 2015;48(3):177–187. 26417477 10.5115/acb.2015.48.3.177 PMC4582160

[79] Sobhani I , Walker F , Roudot‐Thoraval F , Abramowitz L , Johanet H , Henin D , et al. Anal carcinoma: incidence and effect of cumilative infections. AIDS. 2004;18:1561–1569. 15238774 10.1097/01.aids.0000131335.15301.dd

[80] Foster AL , Gaisa MM , Hijdra RM , Turner SS , Morey TJ , Jacobson KB , et al. Shedding of hepatitis C virus into the rectum of HIV‐infected men who have sex with men. Clin Infect Dis. 2017;64(3):284–288. 28013267 10.1093/cid/ciw740

[81] Zhang S , Kodys K , Babcock GJ , Szabo G . CD81/CD9 tetraspanins aid plasmacytoid dendritic cells in recognition of hepatitis C virus‐infected cells and induction of interferon‐alpha. Hepatology. 2013;58(3):940–949. 22577054 10.1002/hep.25827 PMC4511847

[82] Takahashi K , Asabe S , Wieland S , Garaigorta U , Gastaminza P , Isogawa M , et al. Plasmacytoid dendritic cells sense hepatitis C virus‐infected cells, produce interferon, and inhibit infection. Proc Natl Acad Sci USA. 2010;107(16):7431–7436. 20231459 10.1073/pnas.1002301107 PMC2867703

[83] Pawełczyk A , Kubisa N , Jabłońska J , Bukowska‐Ośko I , Caraballo Cortes K , Fic M , et al. Detection of hepatitis C virus (HCV) negative strand RNA and NS3 protein in peripheral blood mononuclear cells (PBMC): CD3 + , CD14 + and CD19 + . Virol J. 2013;10(1):346. 24279719 10.1186/1743-422X-10-346 PMC4222874

[84] Pham TNQ , King D , MacParland SA , McGrath JS , Reddy SB , Bursey FR , et al. Hepatitis C virus replicates in the same immune cell subsets in chronic hepatitis C and occult infection. Gastroenterology. 2008;134(3):812–822. 18243182 10.1053/j.gastro.2007.12.011

[85] Burgess A , Shah K , Hough O , Hynynen K . Investigation of residual hepatitis C virus in presumed recovered subjects. Hepatology. 2013;15(5):477–491. 10.1002/hep.25921 PMC4523271 22729600

[86] Pham TNQ , Michalak TI . Occult persistence and lymphotropism of hepatitis C virus infection. World J Gastroenterol. 2008;14(18):2789–2793. 18473399 10.3748/wjg.14.2789 PMC2710716

[87] Fan H , Qiao L , Kang K‐D , Fan J , Wei W , Luo G . Attachment and postattachment receptors important for hepatitis C virus infection and cell‐to‐cell transmission. J Virol. 2017;91(13):1–20. 10.1128/JVI.00280-17 PMC5469255 28404852

[88] Gardner JP , Durso RJ , Arrigale RR , Donovan GP , Maddon PJ , Dragic T , et al. L‐SIGN (CD 209L) is a liver‐specific capture receptor for hepatitis C virus. Proc Natl Acad Sci USA. 2003;100(8):4498–4503. 12676990 10.1073/pnas.0831128100 PMC153584

[89] Guo Y , Feinberg H , Conroy E , Mitchell DA , Alvarez R , Blixt O , et al. Structural basis for distinct ligand‐binding and targeting properties of the receptors DC‐SIGN and DC‐SIGNR. Nat Struct Mol Biol. 2004;11(7):591–598. 15195147 10.1038/nsmb784

[90] Khoo US , Chan KYK , Chan VSF , Lin CLS . DC‐SIGN and L‐SIGN: the SIGNs for infection. J Mol Med. 2008;86(8):861–874. 18458800 10.1007/s00109-008-0350-2 PMC7079906

[91] Chen Z , Leslie GJ , Lin G , Granelli‐piperno A , Doms RW , Rice CM , et al. Hepatitis C virus glycoproteins interact with DC‐SIGN and DC‐SIGNR. J Virol. 2003;77(7):4070–4080. 12634366 10.1128/JVI.77.7.4070-4080.2003 PMC150620

[92] Cormier EG , Durso RJ , Tsamis F , Boussemart L , Manix C , Olson WC , et al. L‐SIGN (CD209L) and DC‐SIGN (CD209) mediate transinfection of liver cells by hepatitis C virus. Proc Natl Acad Sci USA. 2004;101(39):14067–14072. 15371595 10.1073/pnas.0405695101 PMC521122

[93] Lozach PY , Amara A , Bartosch B , Virelizier JL , Arenzana‐Seisdedos F , Cosset FL , et al. C‐type lectins L‐SIGN and DC‐SIGN capture and transmit infectious hepatitis C virus pseudotype particles. J Biol Chem. 2004;279(31):32035–32045. 15166245 10.1074/jbc.M402296200

[94] Ludwig IS , Lekkerkerker AN , Depla E , Bosman F , Musters RJP , Depraetere S , et al. Hepatitis C virus targets DC‐SIGN and L‐SIGN to escape lysosomal degradation. J Virol. 2004;78(15):8322–8332. 15254204 10.1128/JVI.78.15.8322-8332.2004 PMC446128

[95] Steba GS , Koekkoek SM , Vanhommerig JW , Brinkman K , Kwa D , Van Der Meer JTM , et al. DC‐SIGN polymorphisms associate with risk of hepatitis C virus infection among men who have sex with men but not among injecting drug users. J Infect Dis. 2018;217(3):353–357. 29140443 10.1093/infdis/jix587 PMC5853896

[96] Shi Q , Jiang J , Luo G . Syndecan‐1 serves as the major receptor for attachment of hepatitis C virus to the surfaces of hepatocytes. J Virol. 2013;87(12):6866–6875. 23576506 10.1128/JVI.03475-12 PMC3676102

[97] Falade‐Nwulia O , Chanpimol S , Seamon B , Hernandez H , Harris‐love M , Blackman MR . Oral direct‐acting agent therapy for hepatitis C virus infection: a systematic review. Ann Intern Med. 2017;166(9):637–648. 28319996 10.7326/M16-2575 PMC5486987

[98] European Association for the Study of the Liver . EASL recommendations on treatment of hepatitis C 2018. J Hepatol. 2018;69(2):461–511. 29650333 10.1016/j.jhep.2018.03.026

[99] Martin NK , Thornton A , Hickman M , Sabin C , Nelson M , Cooke GS , et al. Can hepatitis C virus (HCV) direct‐acting antiviral treatment as prevention reverse the HCV epidemic among men who have sex with men in the united kingdom? Epidemiological and modeling insights. Clin Infect Dis. 2016;62(9):1072–1080. 26908813 10.1093/cid/ciw075 PMC4826456

[100] Salazar‐Vizcaya L , Kouyos RD , Zahnd C , Wandeler G , Battegay M , Darling KEA , et al. Hepatitis C virus transmission among human immunodeficiency virus‐infected men who have sex with men: modeling the effect of behavioral and treatment interventions. Hepatology. 2016;64(6):1856–1869. 27531615 10.1002/hep.28769 PMC5132019

[101] Salazar‐Vizcaya L , Wandeler G , Fehr J , Braun D , Cavassini M , Stoeckle M , et al. Impact of direct‐acting antivirals on the burden of HCV infection among persons who inject drugs and men who have sex with men in the Swiss HIV Cohort Study. Open Forum Infect Dis. 2018;5(7):1–4. 10.1093/ofid/ofy154 PMC6047421 30027103

[102] Popping S , Hullegie SJ , Boerekamps A , Rijnders BJA , de Knegt RJ , Rockstroh JK , et al. Early treatment of acute hepatitis C infection is cost‐effective in HIV‐infected men‐who‐have‐sex‐with‐men. PLoS One. 2019;14(1):e0210179. 30629662 10.1371/journal.pone.0210179 PMC6328146

[103] Hill AM , Nath S , Simmons B . The road to elimination of hepatitis C: analysis of cures versus new infections in 91 countries. J virus Erad. 2017;3(3):117–123. 28758018 10.1016/S2055-6640(20)30329-0 PMC5518239

[104] Sacks‐Davis R , Doyle JS , Rauch A , Beguelin C , Pedrana AE , Matthews GV , et al. Linkage and retention in HCV care for HIV‐infected populations: early data from the DAA era. J Int AIDS Soc. 2018;21(S2):e25051. 29633559 10.1002/jia2.25051 PMC5978682

[105] Boerekamps A , van den Berk GE , Lauw FN , Leyten EM , van Kasteren ME , van Eeden A , et al. Declining hepatitis c virus (HCV) incidence in Dutch human immunodeficiency virus‐positive men who have sex with men after unrestricted access to HCV therapy. Clin Infect Dis. 2018;66(9):1360–1365. 29186320 10.1093/cid/cix1007

[106] Price JC , McKinney JE , Crouch P‐C , Dillon SM , Radix A , Stivala A , et al. Sexually acquired hepatitis C infection in HIV‐uninfected men who have sex with men using pre‐exposure prophylaxis against HIV. J Infect Dis. 2018;1–4. 10.1093/infdis/jiy670 30462305

[107] Newsum AM , Stolte IG , Van Der Meer JT , Schinkel J , Van Der Valk M , Vanhommerig JW , et al. Development and validation of the HCV‐MOSAIC risk score to assist testing for acute hepatitis C virus (HCV) infection in HIV‐infected men who have sex with men (MSM). Eurosurveillance. 2017;22(21):30540. 28597832 10.2807/1560-7917.ES.2017.22.21.30540 PMC5479984

[108] Volz EM , Ionides E , Romero‐Severson EO , Brandt M‐G , Mokotoff E , Koopman JS . HIV‐1 transmission during early infection in men who have sex with men: a phylodynamic analysis. PLoS Med. 2013;10(12):e1001568. 24339751 10.1371/journal.pmed.1001568 PMC3858227

[109] Chung RT , Davis GL , Jensen DM , Masur H , Saag MS , Thomas DL , et al. Hepatitis C guidance: AASLD‐IDSA recommendations for testing, managing, and treating adults infected with hepatitis C virus. Hepatology. 2015;62(3):932–954. 26111063 10.1002/hep.27950

[110] Chung RT , Ghany MG , Kim AY , Marks KM , Naggie S , Vargas HE , et al. Hepatitis C guidance 2018 update: AASLD‐IDSA recommendations for testing, managing, and treating Hepatitis C Virus infection. Clin Infect Dis. 2018;67(10):1477–1492. 30215672 10.1093/cid/ciy585 PMC7190892

[111] Vogel M , Deterding K , Wiegand J , Grüner NH , Baumgarten A , Jung MC , et al. Initial presentation of acute hepatitis C virus (HCV) infection among HIV–negative and HIV‐positive individuals – experience from 2 large German networks on the study of acute HCV infection. Clin Infect Dis. 2009 Jul;49(2):317–319. 19538070 10.1086/600058

[112] Vanhommerig JW , Thomas XV , van der Meer JTM , Geskus RB , Bruisten SM , Molenkamp R , et al. Hepatitis C virus (HCV) antibody dynamics following acute HCV infection and reinfection among HIV‐infected men who have sex with men. Clin Infect Dis. 2014;59(12):1678–1685. 25186590 10.1093/cid/ciu695

[113] Thomson EC , Nastouli E , Main J , Karayiannis P , Eliahoo J , Muir D , et al. Delayed anti‐HCV antibody response in HIV‐positive men acutely infected with HCV. AIDS. 2009 Jan;23(1):89–93. 19050390 10.1097/QAD.0b013e32831940a3 PMC2646374

[114] Vanhommerig JW , Schinkel J , Van Der Valk M . Seven years of chronic hepatitis C virus infection in an HIV‐infected man without detectable antibodies. AIDS. 2014;29(3):389–394. 10.1097/QAD.0000000000000541 25686686

[115] Chamie G , Bonacini M , Bangsberg DR , Stapleton JT , Hall C , Overton ET , et al. Factors associated with seronegative chronic hepatitis C virus infection in HIV infection. Clin Infect Dis. 2007;44(4):577–583. 17243063 10.1086/511038 PMC3170414

[116] Aids. The European AIDS Treatment Network (NEAT) acute hepatitis C infection consensus panel. Acute hepatitis c in HIV‐infected individuals: recommendations from the European Aids Treatment Network (NEAT) consensus conference. 2011;25(4):399–409. 21139491 10.1097/QAD.0b013e328343443b

[117] van Tilborg M , Al Marzooqi SH , Wong WWL , Maan R , Vermehren J , Maasoumy B , et al. HCV core antigen as an alternative to HCV RNA testing in the era of direct‐acting antivirals: retrospective screening and diagnostic cohort studies. Lancet Gastroenterol Hepatol. 2018;3(12):856–864. 30274834 10.1016/S2468-1253(18)30271-1

[118] Cresswell FV , Fisher M , Hughes DJ , Shaw SG , Homer G , Ibrahim MO . Hepatitis C core antigen testing: a reliable, quick, and potentially cost‐effective alternative to hepatitis C polymerase chain reaction in diagnosing acute hepatitis C virus infection. Clin Infect Dis. 2015;60(2):263–266. 25301216 10.1093/cid/ciu782

[119] Vanhommerig JW , van de Laar TJW , Koot M , van Rooijen MS , Schinkel J , Speksnijder AGCL , et al. Evaluation of a hepatitis C virus (HCV) antigen assay for routine HCV screening among men who have sex with men infected with HIV. J Virol Methods. 2015;213:147–150. 25528203 10.1016/j.jviromet.2014.11.026

[120] Fisher DG , Hess KL , Erlyana E , Reynolds GL , Cummins CA , Alonzo TA . Comparison of rapid point‐of‐care tests for detection of antibodies to hepatitis C virus. Open Forum Infect Dis. 2015;2(3):101. 10.1093/ofid/ofv101 PMC4531224 26269795

[121] Lamoury FMJ , Hajarizadeh B , Soker A , Martinez D , Quek C , Cunningham P , et al. Evaluation of a hepatitis C virus core antigen assay in plasma and dried blood spot samples. J Mol Diagn. 2018;20(5):621–627. 29959023 10.1016/j.jmoldx.2018.05.010

[122] Nguyen TT , Lemee V , Bollore K , Vu HV , Lacombe K , Thi XLT , et al. Confirmation of HCV viremia using HCV RNA and core antigen testing on dried blood spot in HIV infected peoples who inject drugs in Vietnam. BMC Infect Dis. 2018;18(1):622. 30514229 10.1186/s12879-018-3529-3 PMC6280470

[123] Braun DL , Marzel A , Steffens D , Schreiber PW , Grube C , Scherrer AU , et al. High rates of subsequent asymptomatic sexually transmitted infections and risky sexual behavior in patients initially presenting with primary human immunodeficiency virus‐1 infection. Clin Infect Dis. 2018;66(5):735–742. 29028966 10.1093/cid/cix873

[124] Kaplan‐Lewis E , Fierer DS . Acute HCV in HIV‐infected MSM: modes AOF acquisition, liver fibrosis, and treatment. Curr HIV/AIDS Rep. 2015;12(3):317–325. 26152661 10.1007/s11904-015-0279-3

